# Supplementary material for: The genetic architecture of floral traits in the woody plant Prunus mume
Source: Nat Commun. 2018 Apr 27;9:1702. doi: 10.1038/s41467-018-04093-z (PMC5923208; doi:10.1038/s41467-018-04093-z)
Supplement: Supplementary file 1 — Supplementary Information [file 41467_2018_4093_MOESM1_ESM.pdf]

SUPPLEMENTARY INFORMATION

Zhang et al.

The genetic architecture of floral traits in the woody plant *Prunus mume*

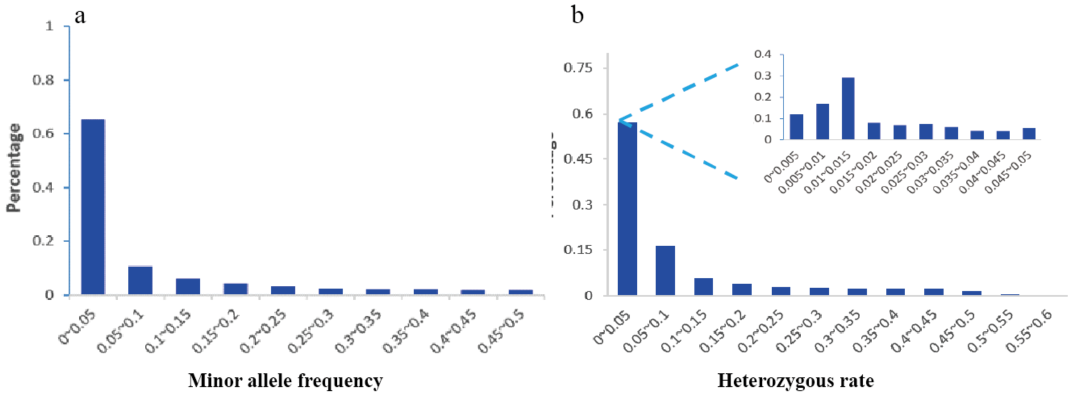

Supplementary Figure 1. Statistical information of population SNP of mei. a. Minor allele frequency spectrum. **b.** proportion of heterozygous genotype.

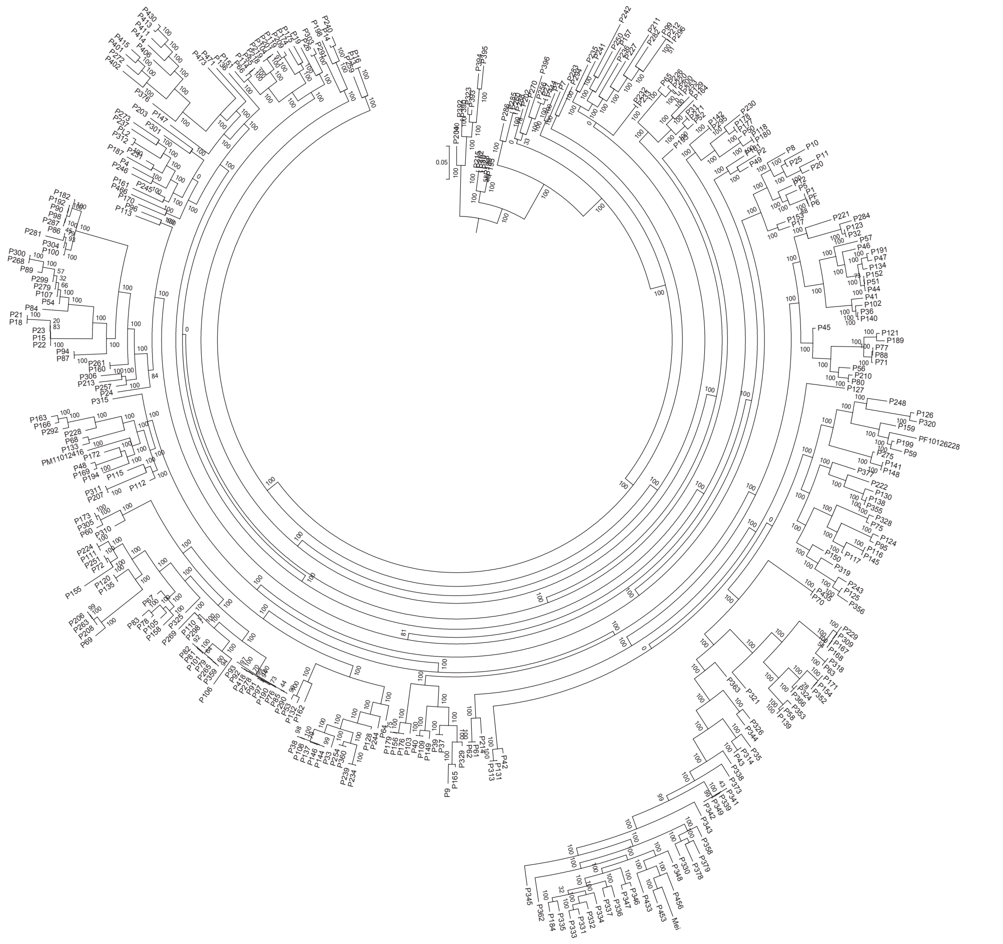

Supplementary Figure 2. Phylogenetic tree of 351 *Prunus* individuals using genome-wide SNPs.

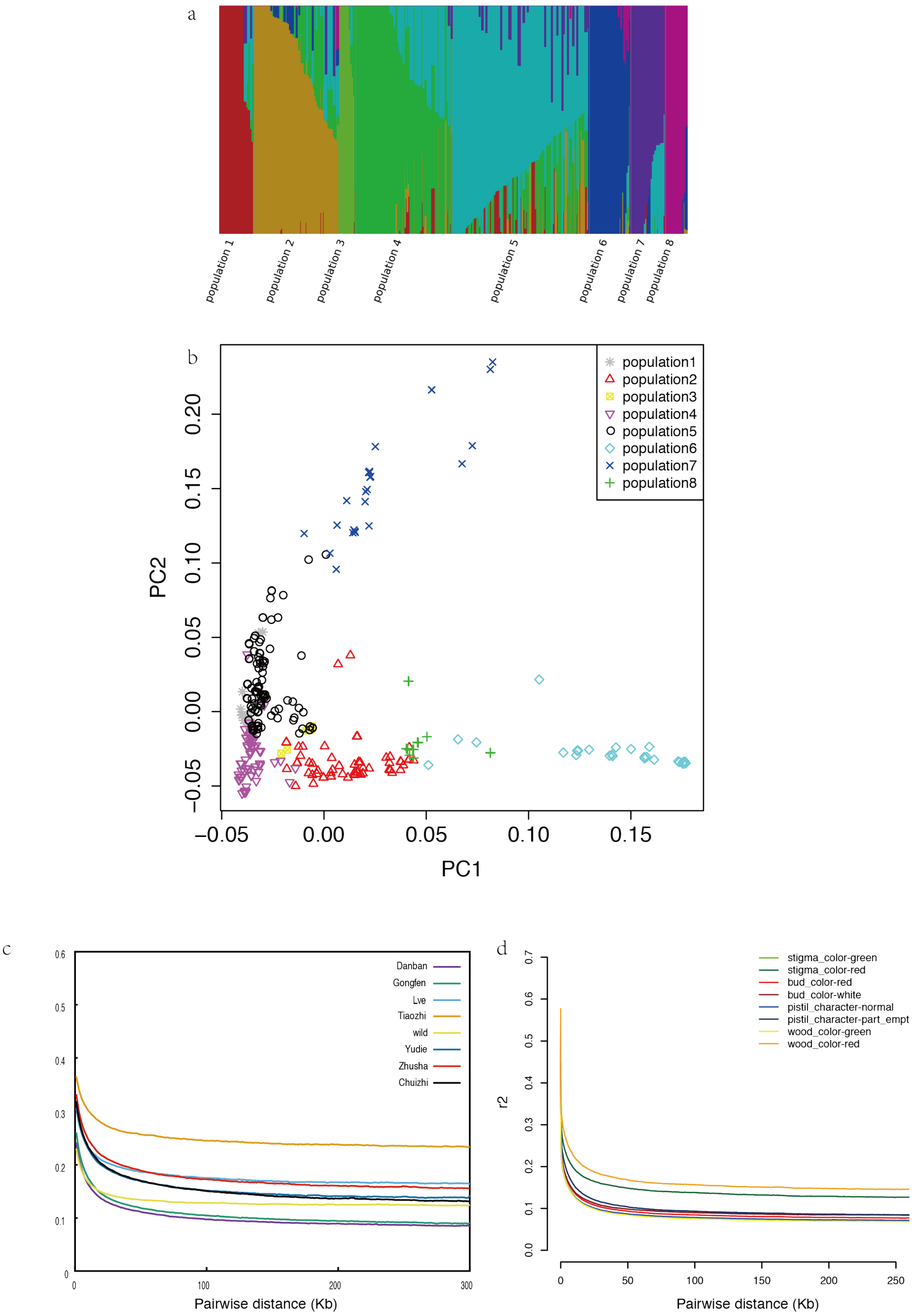

Supplementary Figure 3. Population structure and decay of LD in mei populations. a. Model-based cluster analysis of population structures represented by different numbers of clusters (k = 8). The *y*-axis denotes cluster membership and the *x*-axis denotes each accession. **b.** PCA of 351 *Prunus* individuals using genome-wide SNPs. **c.** Linkage disequilibrium difference among wild and different cultivar classes of mei. **d.** Linkage disequilibrium differences between different phenotypes for each trait including the colors of wood, stigma, and bud, and pistil character. The light and dark green line represents samples with green and red stigmas; the red and brown line represent samples with red or white bud color; the blue and purple line represent samples with normal or partially absent pistil; the yellow and orange line represent samples with green or red wood.

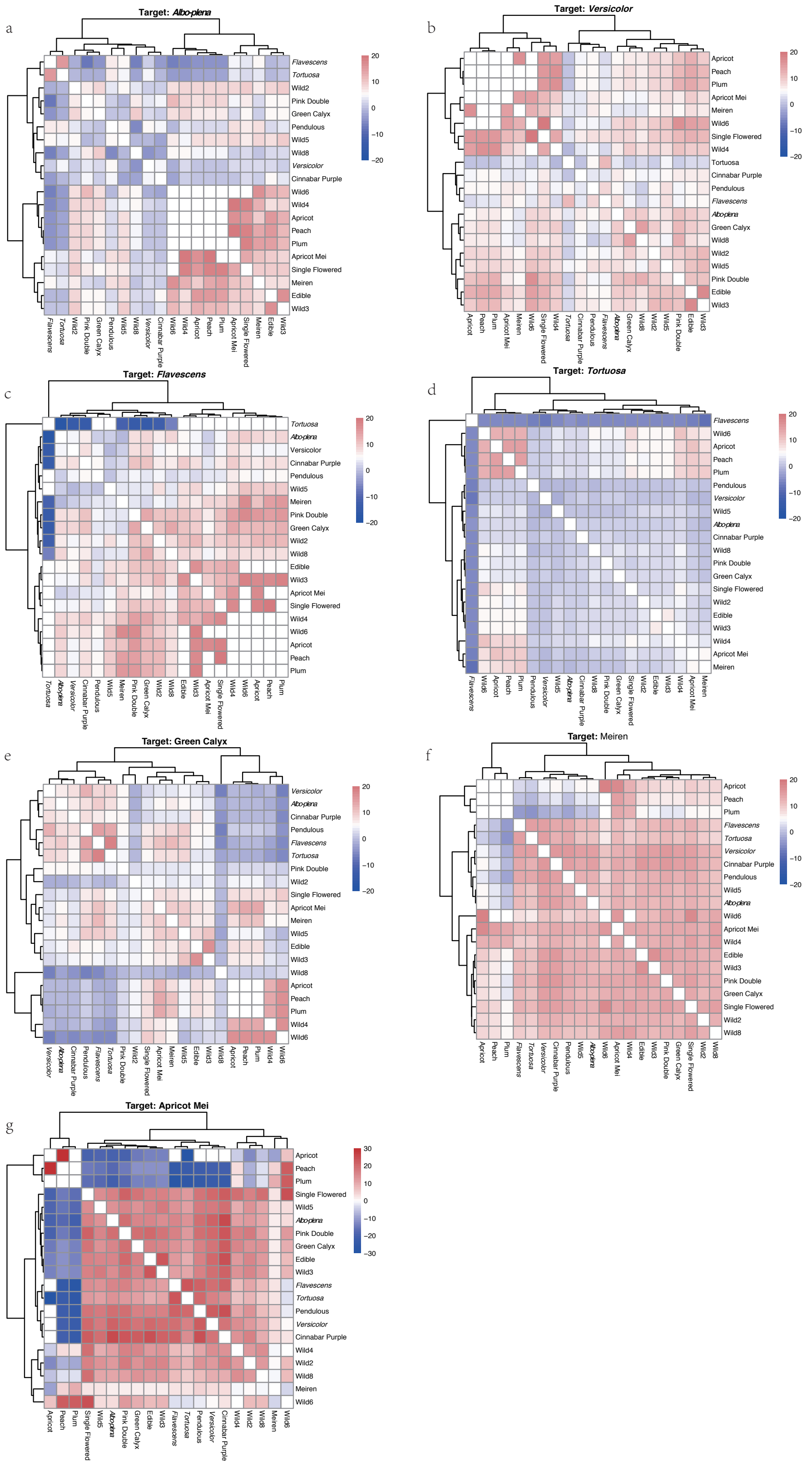

Supplementary Figure 4. Introgression in different cultivar groups. Heat map for introgression analysis using three-population F3 test statistics. **a-g** denote target population of *Albo-plena*, *Versicolor*, *Flavescens*, *Tortuosa*, Green Calyx, Meiren and Apricot Mei, respectively.

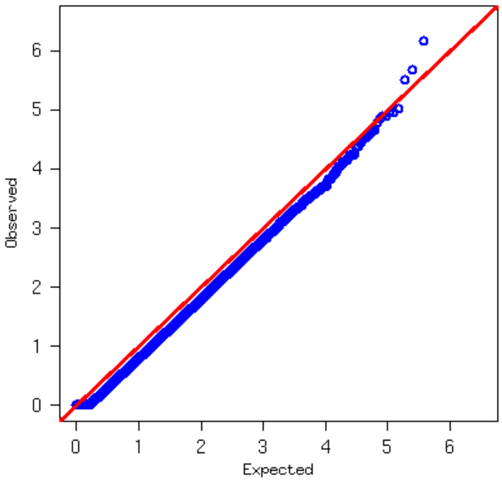
Observed
Expected

a

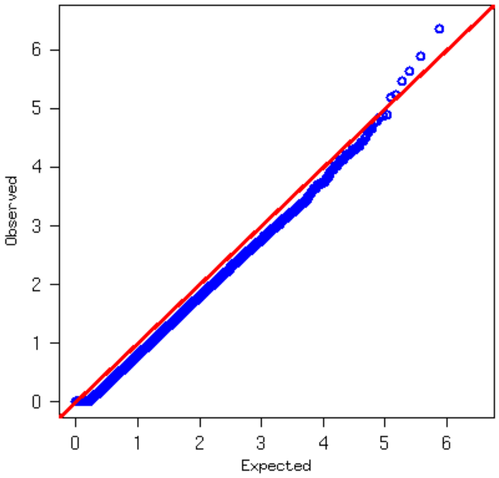
Observed
Expected

b

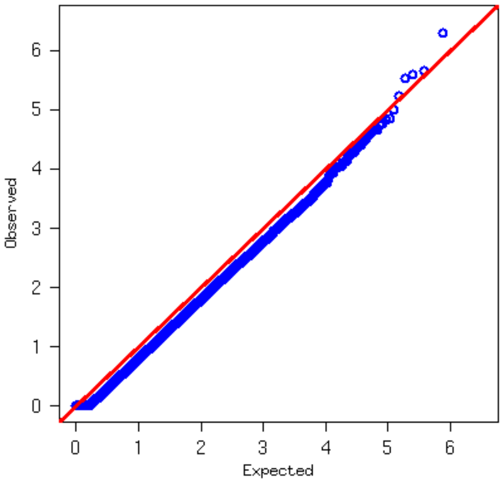
Observed
Expected

c

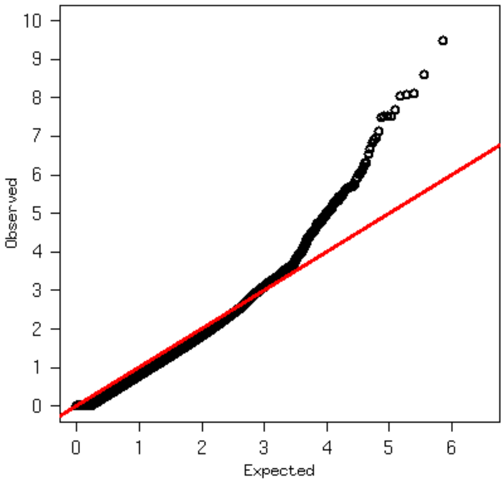
Observed
Expected

d

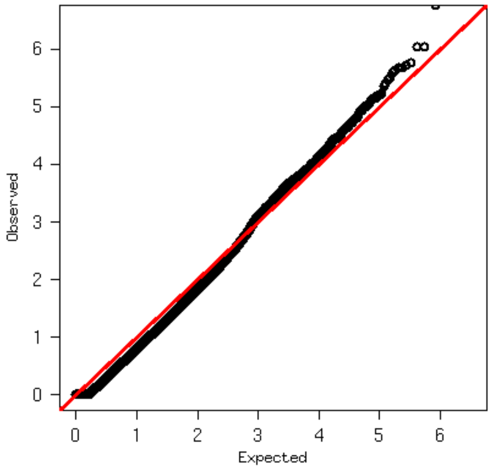
Observed
Expected

e

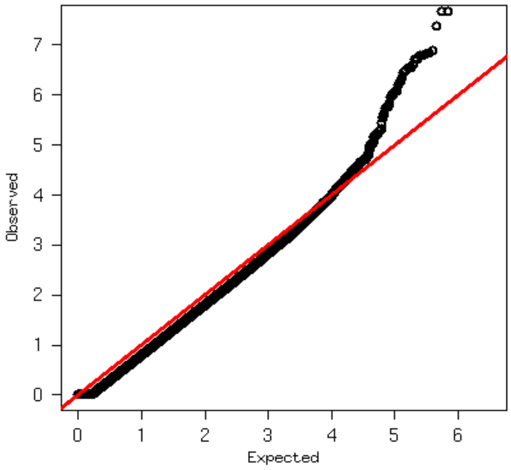
Observed
Expected

f

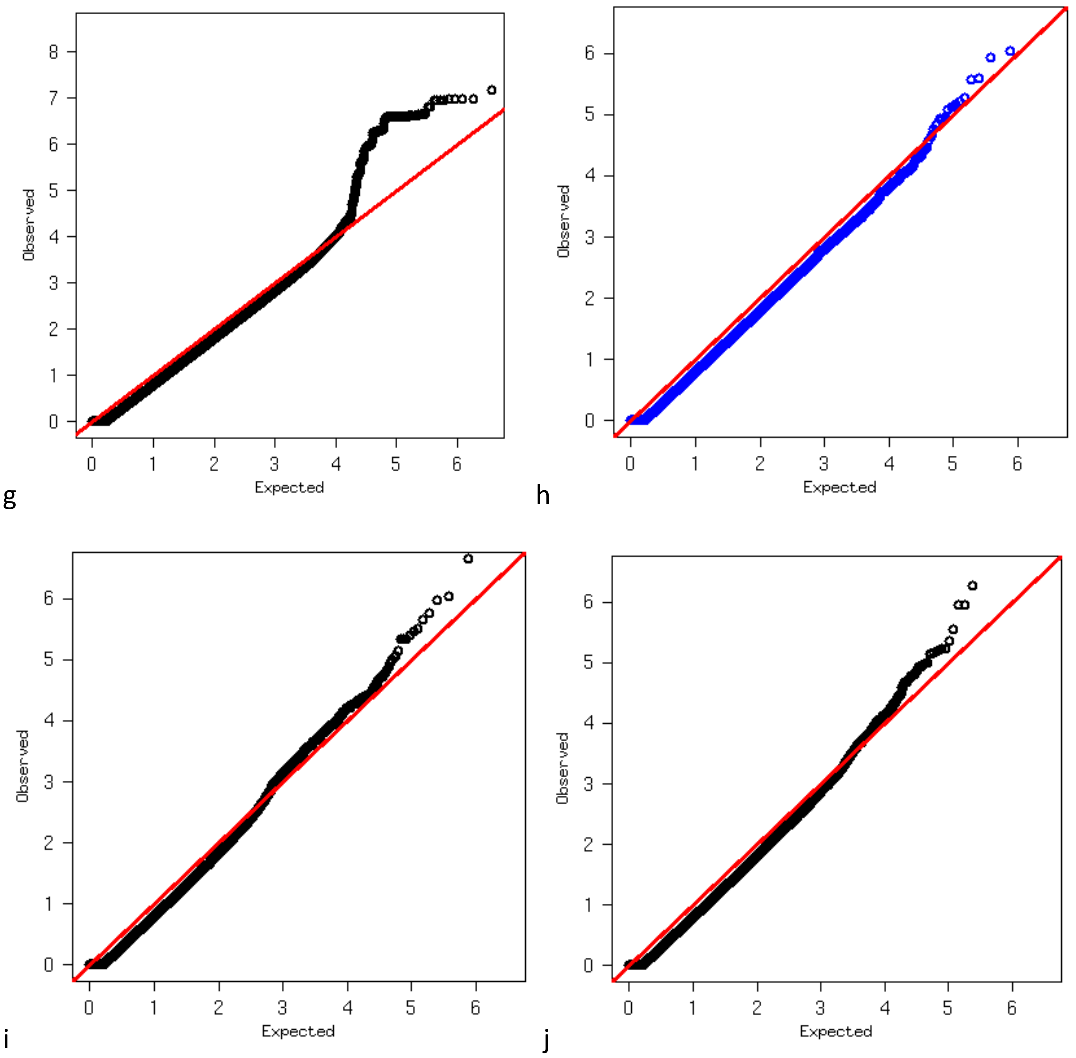

Supplementary Figure 5. Q-Q plots for GWAS p-values of 10 traits. **a-j** denote results of petal number, petal color, stigma color, bud color, wood color, staminal filament color, pistil character, bud aperture, branching phenotype, calyx color, respectively. Blue and black dots represent different optimal model (Q+K and Q) chosen for each trait.

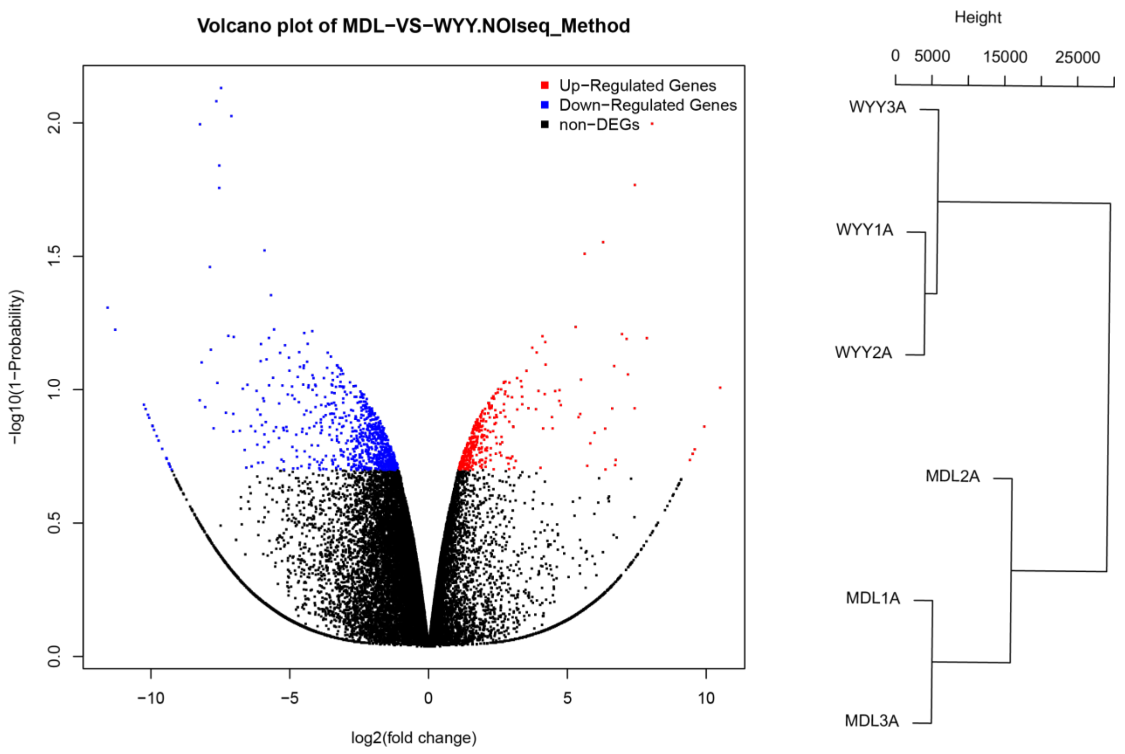

a b

Supplementary Figure 6. Identification of differentially expressed genes (DEGs) and cluster analysis of six samples. a. DEGs and SEGs identified between the samples with white flowers (MDL) and samples with red flowers (WYY). Blue dots represent down-regulated genes and red dots represent up-regulated genes. **b.** Clustering of these six samples (three replicates of MDL and three replicates of WYY) based on all DEGs.

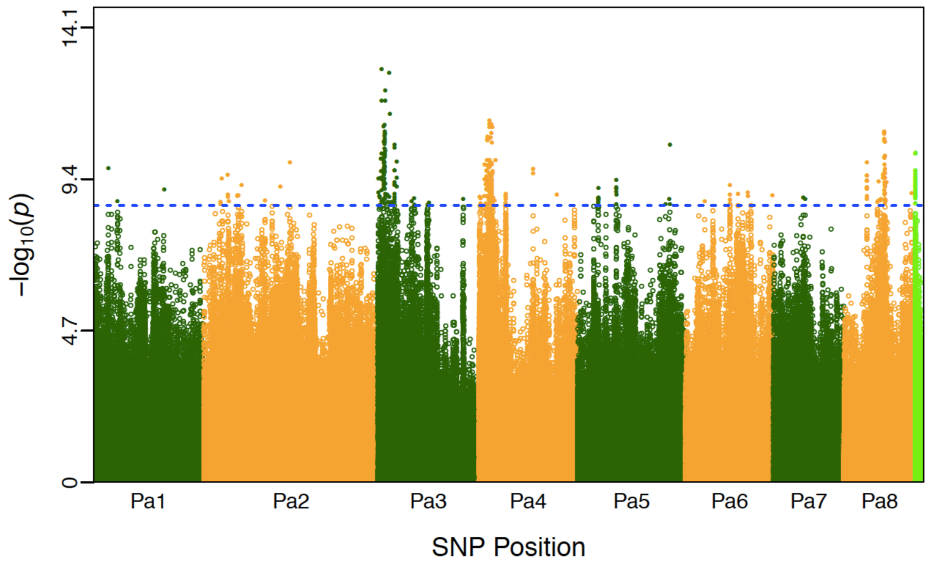

Supplementary Figure 7. GWAS results for calyx color in mei. Manhattan plot of negative log-likelihood ratio of the extent to which each SNP is associated with calyx color across the *P. mume* genome. Dark green dots represent SNPs on chromosomes Pa1, Pa3, Pa5 and Pa7; orange dots represent SNPs on chromosomes Pa2, Pa4, Pa6 and Pa8; and light green dots represent SNPs in scaffolds not anchored to any chromosome. Pa1–8 represent the chromosomes of the reference genome *Prunus mume*).

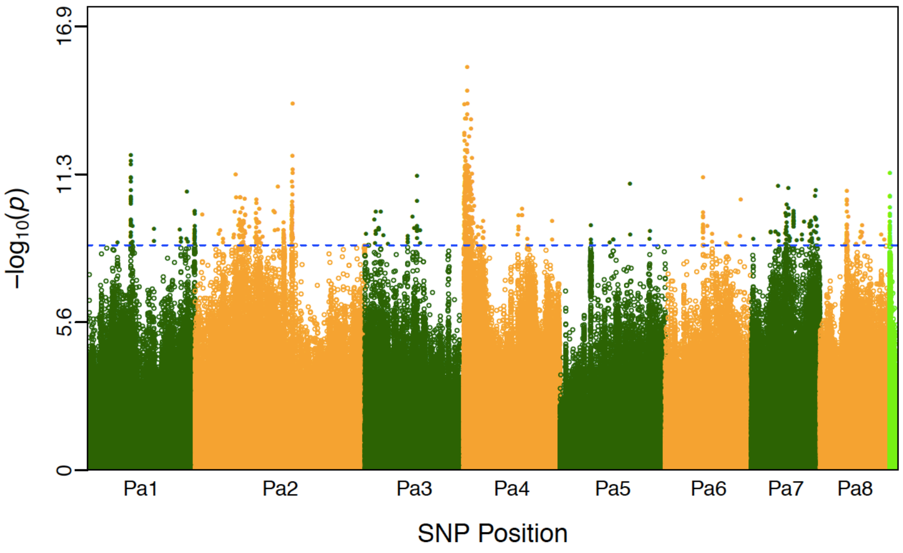

Supplementary Figure 8. GWAS results of bud color in mei. Manhattan plot of negative log-likelihood ratio of the extent to which each SNP is associated with bud color across the *P. mume* genome. Dark green dots represent SNPs on chromosomes Pa1, Pa3, Pa5 and Pa7; orange dots represent SNPs on chromosomes Pa2, Pa4, Pa6 and Pa8; and light green dots represent SNPs in scaffolds not anchored to any chromosome. Pa1–8 represent the chromosomes of the reference genome *Prunus mume*).

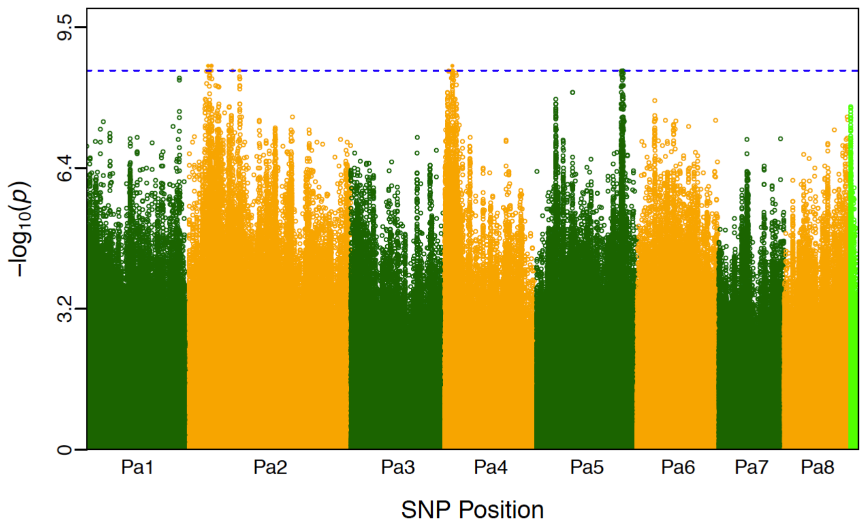

Supplementary Figure 9. GWAS results of stigma color in mei. Manhattan plot of negative log-likelihood ratio of the extent to which each SNP is associated with stigma color across the *P. mume* genome. Dark green dots represent SNPs on chromosomes Pa1, Pa3, Pa5 and Pa7; orange dots represent SNPs on chromosomes Pa2, Pa4, Pa6 and Pa8; and light green dots represent SNPs in scaffolds not anchored to any chromosome. Pa1–8 represent the chromosomes of the reference genome *Prunus mume*).

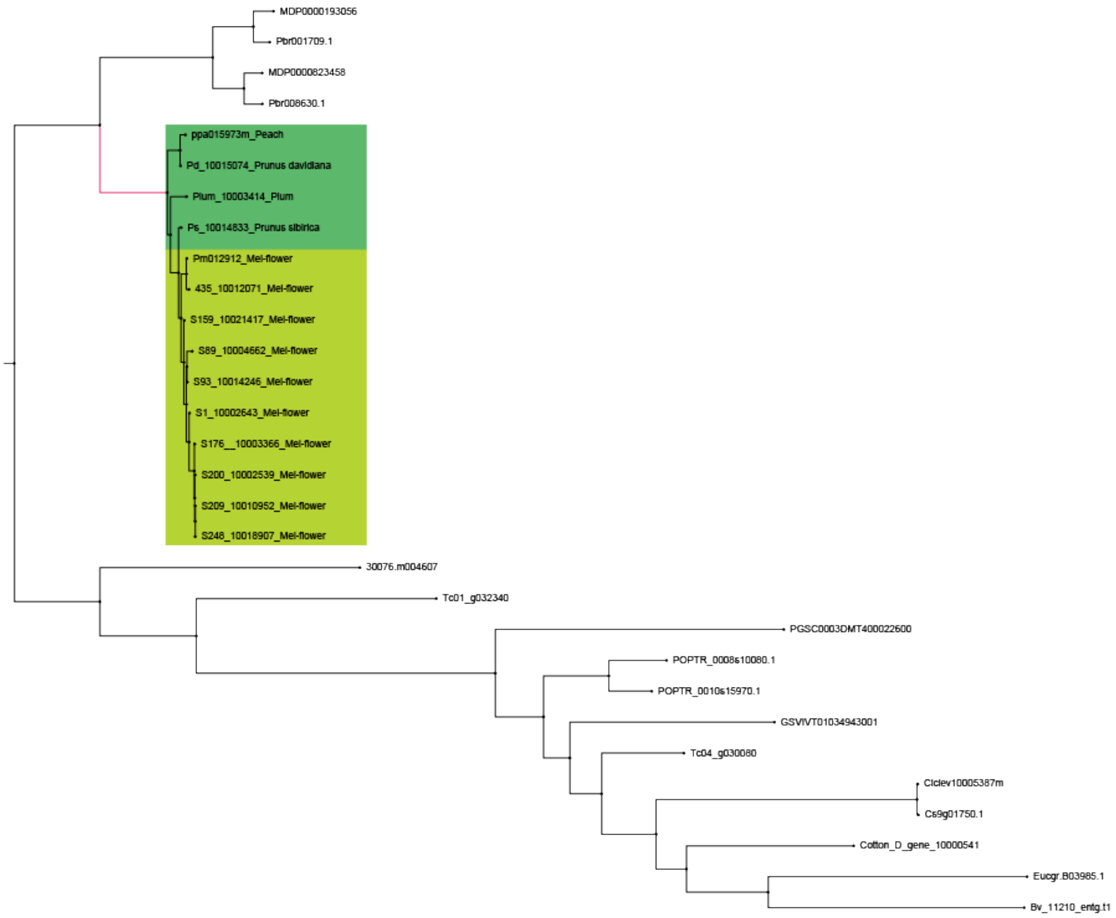

Supplementary Figure 10. Phylogenetic tree of *MYB108* genes in *Prunus*. A phylogenetic tree constructed from *MYB108* sequences in *Prunus* species including *P. domestica*, *P. davidiana*, *P. sibirica* and *P. persica*, and other non-*Prunus* species as outgroups.

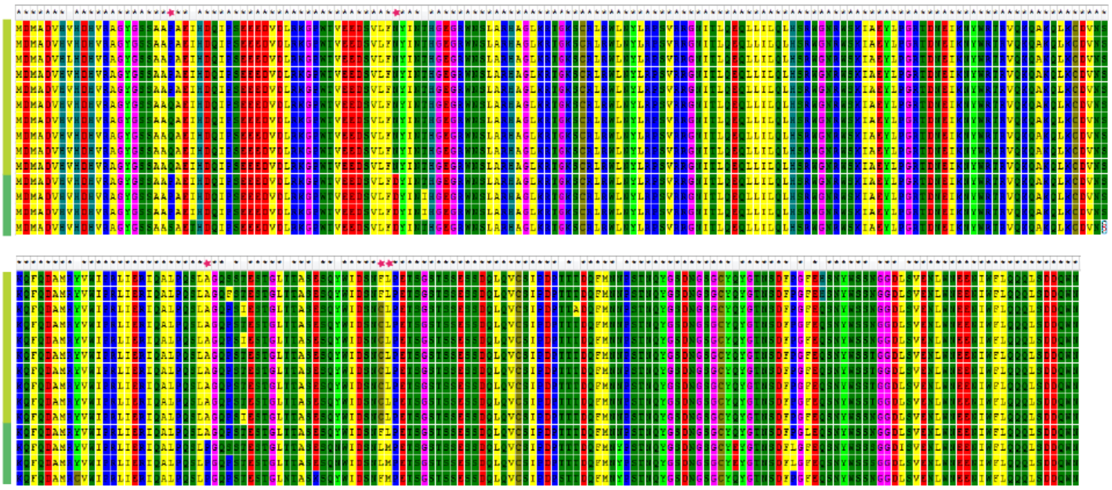

Supplementary Figure 11. Comparison of amino acid sequences of MYB108 from *Prunus*. Multiple sequence alignment of the MYB108 protein sequence *Prunus* species (including *P. domestica*, *P. davidiana*, *P. sibirica* and *P. persica*) and eight core pan-genome mei samples was performed. *MYB108* is highly conserved within *Prunus*, but differs notably among clades. Each amino acid is represented by a different color.

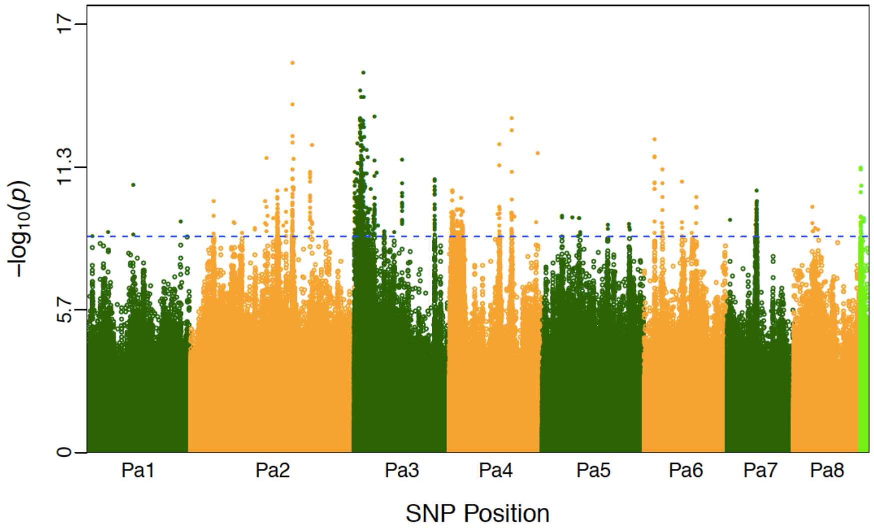

Supplementary Figure 12. GWAS results for staminal filament color in mei. Manhattan plot of negative log-likelihood ratio of the extent to which each SNP is associated with staminal filament color across the *P. mume* genome. Dark green dots represent SNPs on chromosomes Pa1, Pa3, Pa5 and Pa7; orange dots represent SNPs on chromosomes Pa2, Pa4, Pa6 and Pa8; and light green dots represent SNPs in scaffolds not anchored to any chromosome. Pa1–8 represent the chromosomes of the reference genome *Prunus mume*).

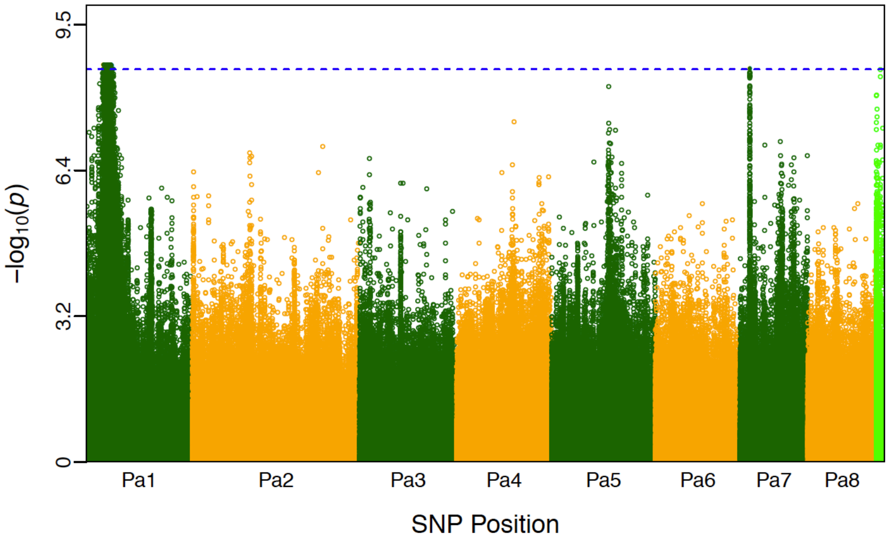

Supplementary Figure 13. GWAS results for bud aperture in mei. Manhattan plot of negative log-likelihood ratio of the extent to which each SNP is associated with bud aperture across the *P. mume* genome. Dark green dots represent SNPs on chromosomes Pa1, Pa3, Pa5 and Pa7; orange dots represent SNPs on chromosomes Pa2, Pa4, Pa6 and Pa8; and light green dots represent SNPs in scaffolds not anchored to any chromosome. Pa1–8 represent the chromosomes of the reference genome *Prunus mume*).

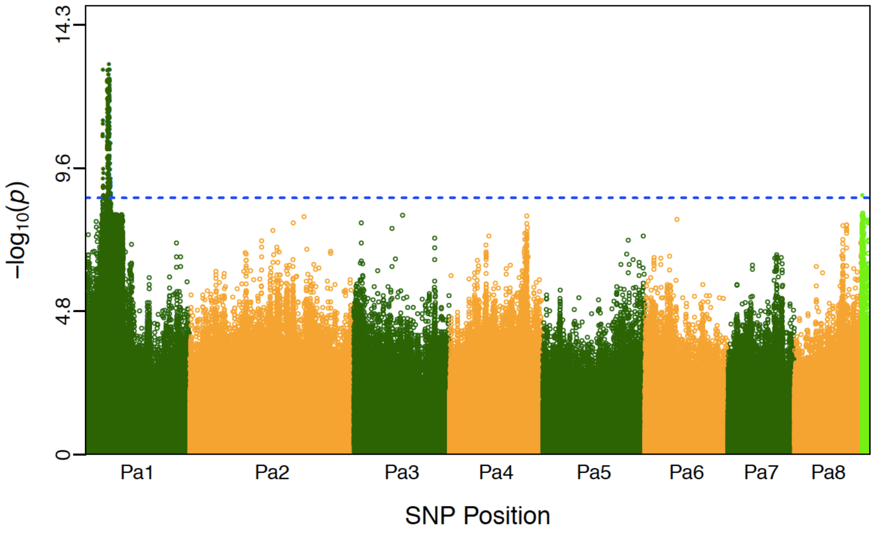

Supplementary Figure 14. GWAS results for the pistil character in mei. Manhattan plot of negative log-likelihood ratio of the extent to which each SNP is associated with the pistil character across the *P. mume* genome. Dark green dots represent SNPs on chromosomes Pa1, Pa3, Pa5 and Pa7; orange dots represent SNPs on chromosomes Pa2, Pa4, Pa6 and Pa8; and light green dots represent SNPs in scaffolds not anchored to any chromosome. Pa1–8 represent the chromosomes of the reference genome *Prunus mume*).

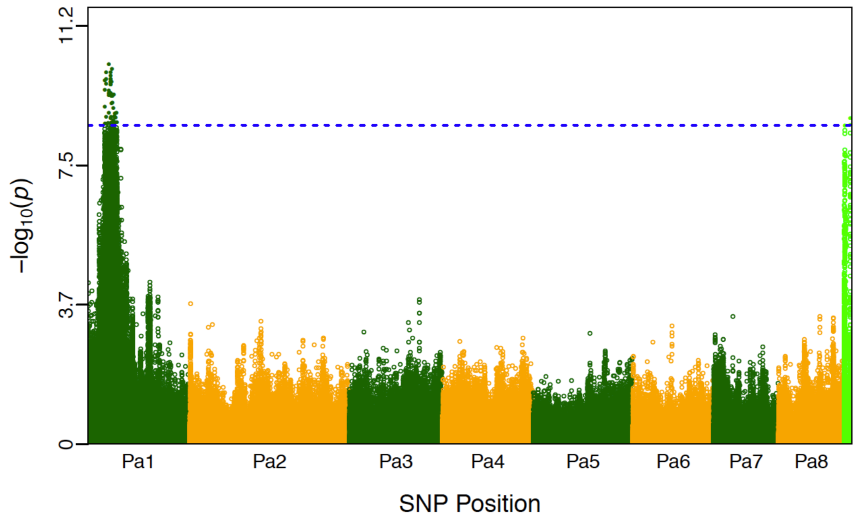

Supplementary Figure 15. GWAS results for petal number in mei. Manhattan plot the negative log-likelihood ratio of the extent to which each SNP is associated with petal number on the eight chromosomes of mei flower. Dark green dots represent SNPs on chromosomes Pa1, Pa3, Pa5 and Pa7; orange dots represent SNPs on chromosomes Pa2, Pa4, Pa6 and Pa8; and light green dots represent SNPs in scaffolds not anchored to any chromosome. Pa1–8 represent the chromosomes of the reference genome *Prunus mume*).

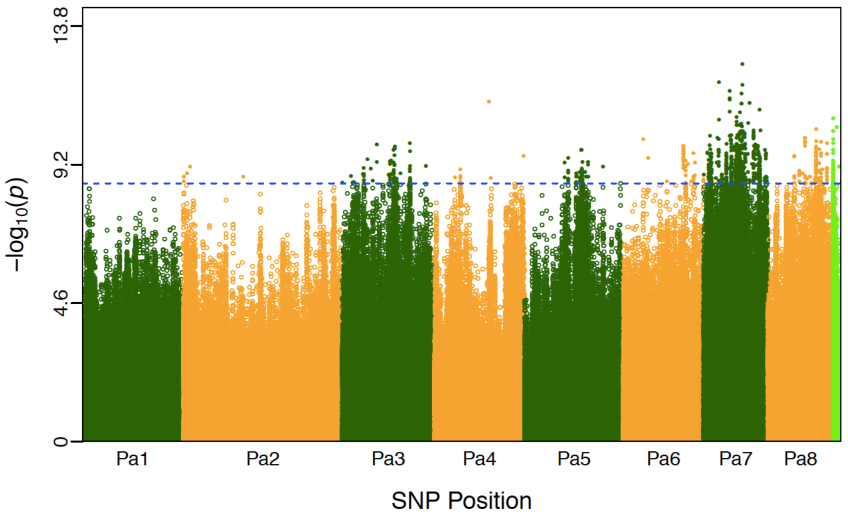

Supplementary Figure 16. GWAS results for branch phenotype in mei. Manhattan plot of negative log-likelihood ratio of the extent to which each SNP is associated with branching phenotype across the *P. mume* genome. Dark green dots represent SNPs on chromosomes Pa1, Pa3, Pa5 and Pa7; orange dots represent SNPs on chromosomes Pa2, Pa4, Pa6 and Pa8; and light green dots represent SNPs in scaffolds not anchored to any chromosome. Pa1–8 represent the chromosomes of the reference genome *Prunus mume*).

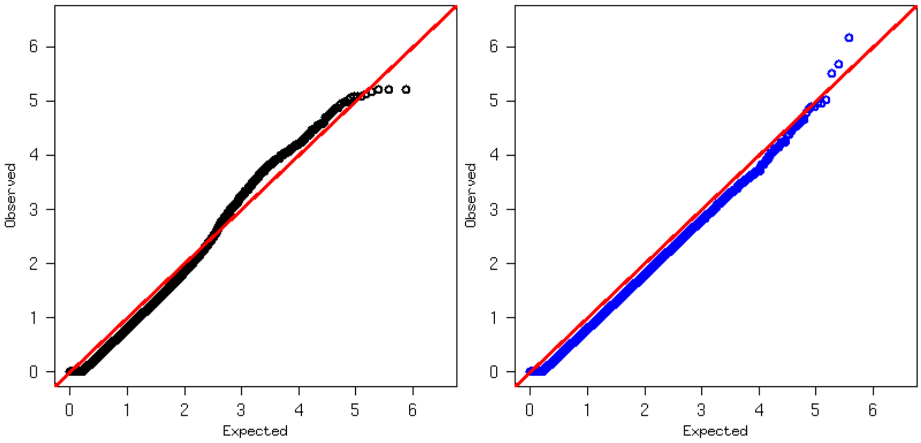

Petal number

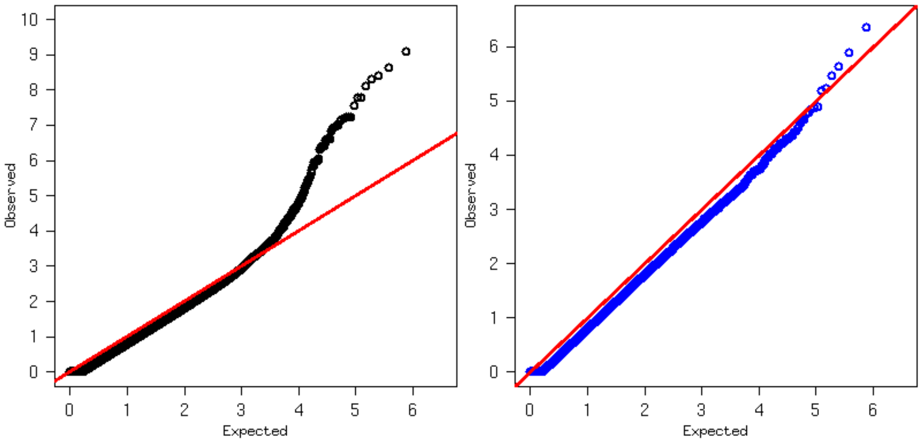

Petal color

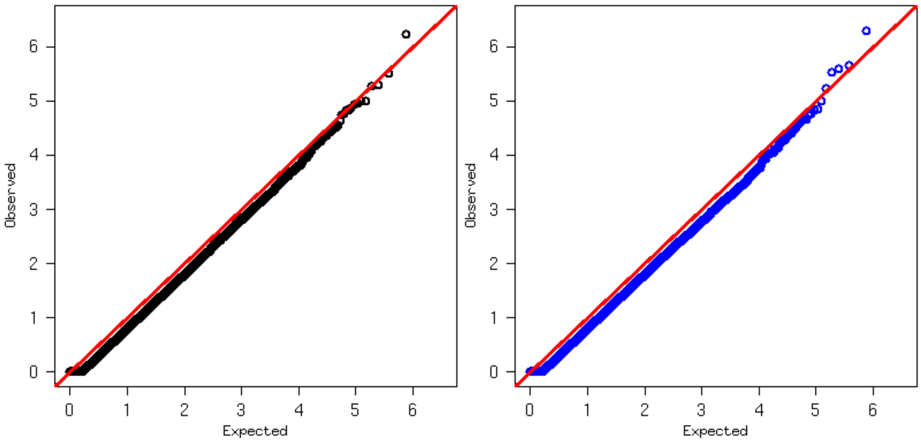

Stigma color

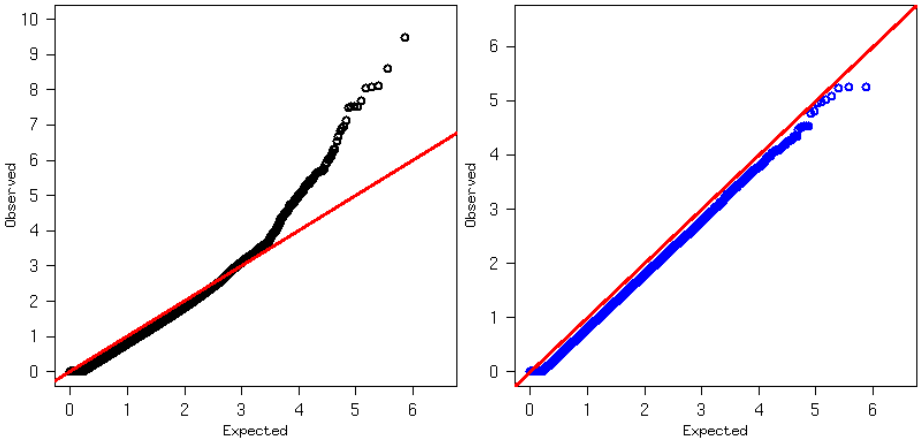

Bud color

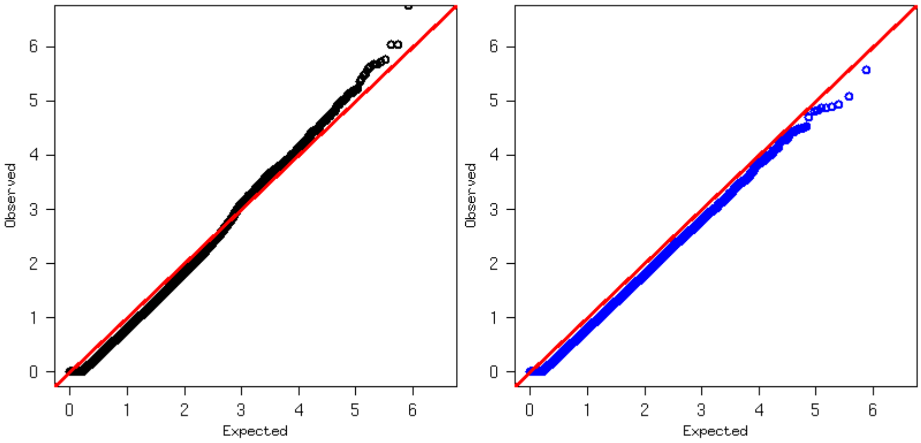

Wood color

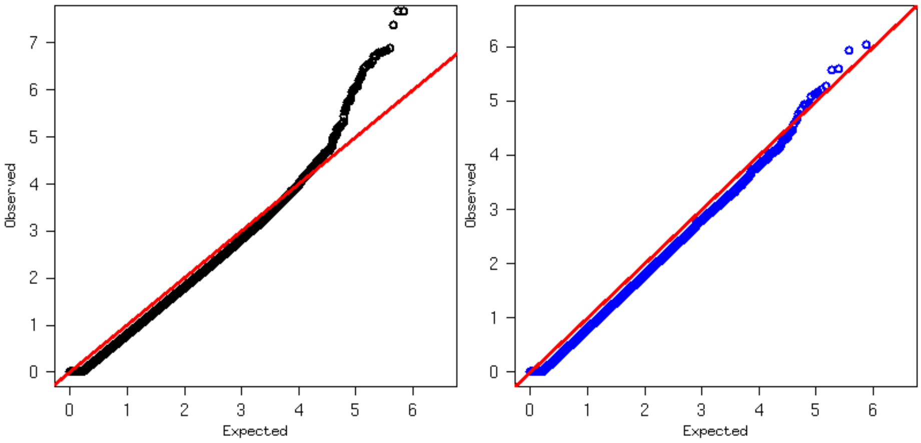

Staminal filament color

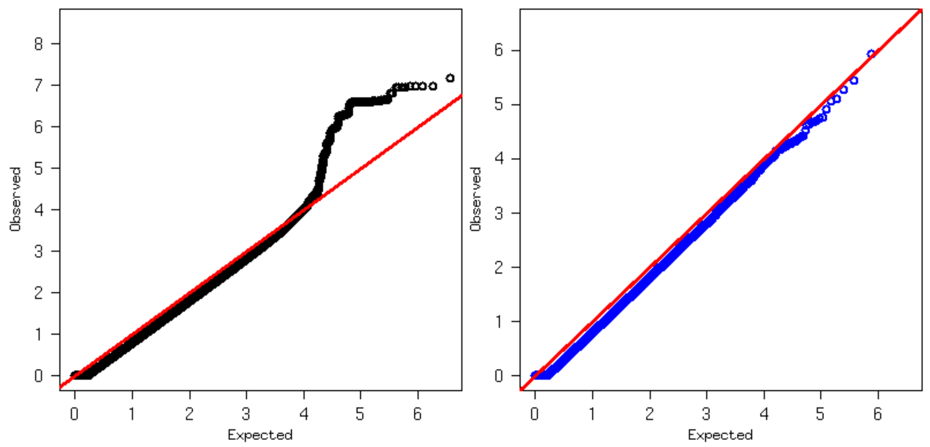

Pistil character

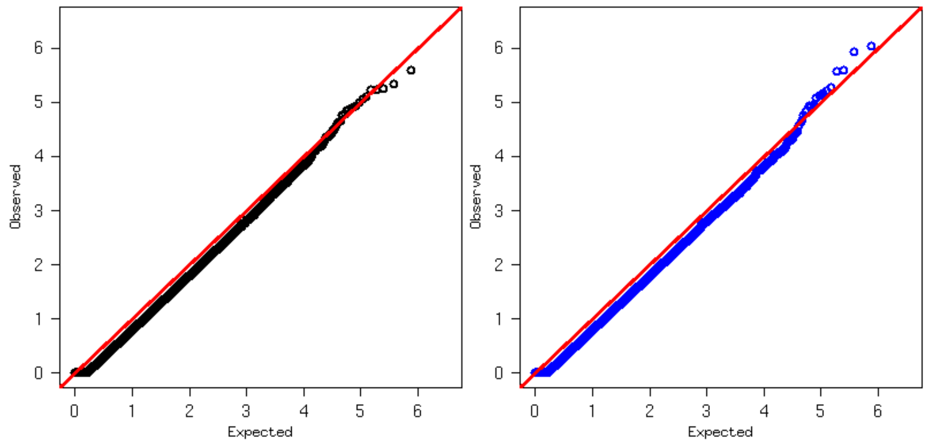

Bud aperture

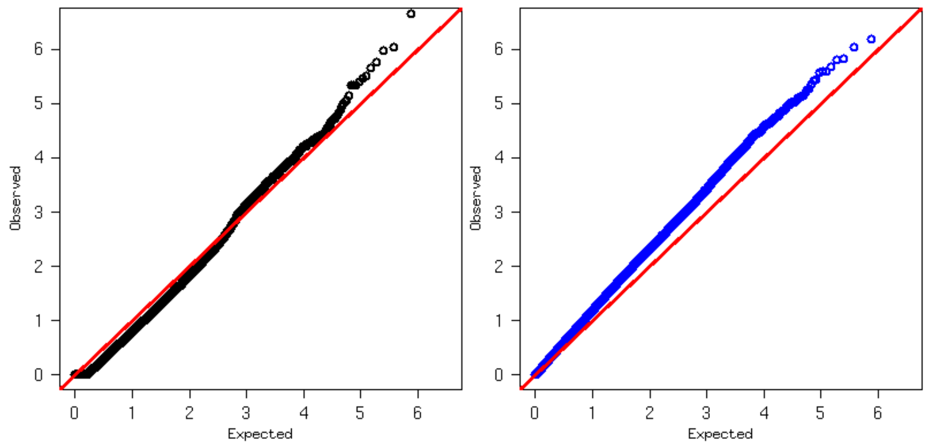

Branch phenotype

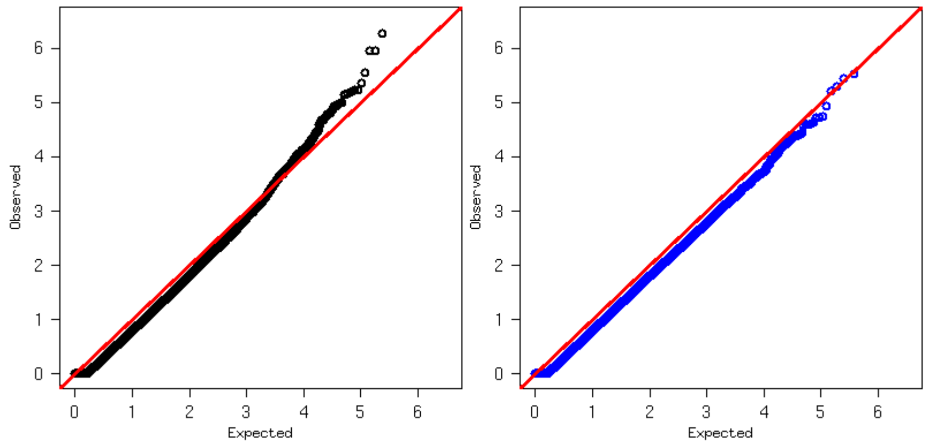

Calyx color

Supplementary Figure 17. Q-Q plot of both Q and Q+K model for each trait.

Black and blue plots represented Q and Q+K model respectively.

Supplementary Table 1. Summary of SNP identification in re-sequenced population*

Chr.**	Total No. of SNPs	Intergenic	Intragenic					
			Intron		CDS			
			Intron	Splice intron	Synonymous	Non-synonymous	
						Missense	Nonsense
Pa 1	1,586,915	1,153,025	274,908	562	71,775	88,371	2,343
Pa 2	1,405,115	1,733,652	484,303	783	111,109	132,973	3,389
Pa 3	2,459,711	1,001,770	257,496	528	61,844	82,765	2,311
Pa 4	1,411,588	1,002,139	262,282	411	65,997	81,187	2,078
Pa 5	1,489,025	1,054,040	279,754	516	68,310	88,575	2,387
Pa 6	1,227,443	860,831	237,857	482	56,467	74,938	2,028
Pa 7	956,489	678,433	180,124	305	44,568	53,526	1,361
Pa 8	1,040,788	744,457	188,244	319	48,788	59,702	1,459
Pa 0	1,187,268	994,966	102,987	734	36,046	50,276	3,623
Total	12,764,342	9,223,313	2,267,955	4,640	564,904	712,313	20,979

*348 cultivated and wild *Prunus mume* individuals and three close relatives of mei, *P. sibirica*, *P. davidiana* and *P. salicina*; **Chromosome numbers correspond to those in *P. armeniaca*

Supplementary Table 2. Comparison of genomic diversity in major crops

**Lines**	Population	Lines	Average genome coverage	Diversity(π)	Publication
Cucumber	W*	30	16.7×	0.0045	Qi et al., 2013
	C*	85	18.9×	0.0023	
Watermelon	W	10	4.1×	0.0076	Guo et al., 2013
	C	10	10.4×	0.0014	
Tomato	W	53	5.7×	0.0032	Lin et al., 2014
	C	112	5.7×	0.0017	
Rice	W	446	1.97×	0.003	Huang et al., 2012
	C	1,083	0.89×	0.0024	
Maize	W	17	4.7×	0.0059	Hufford et al., 2012
	C	23	5.3×	0.0048	
Soybean	W	62	11×	0.0029	Zhou et al., 2015
	C	110	11×	0.001	
Cotton	W	30	6.2×	0.0013	Wang et al. 2017
	C	267	6.2×	0.0007	
Mei	W	15	19.3×	0.0028	This study
	C	303	19.3×	0.0020	

Supplementary Table 3. Sequence depth distribution and the relation of GC frequency and depth average of each genome assembly

Sanple ID	Sequence Depth	GC Depth
S93	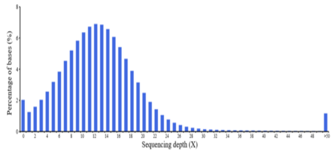	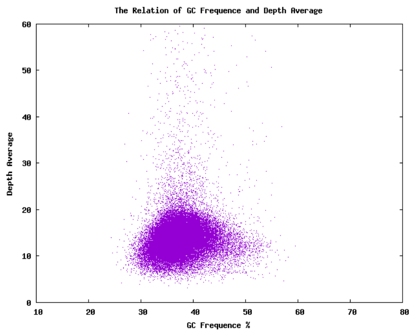
435	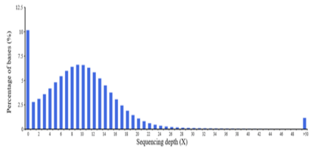	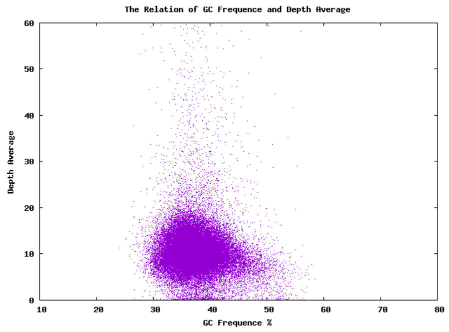
S89	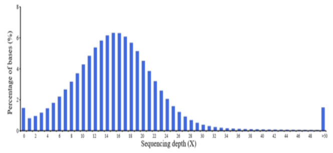	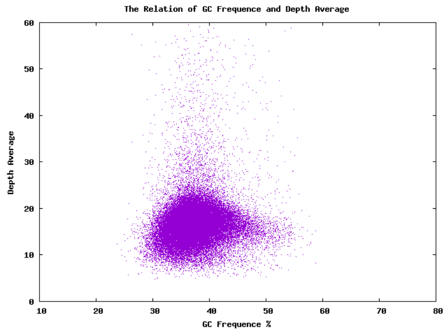
S1	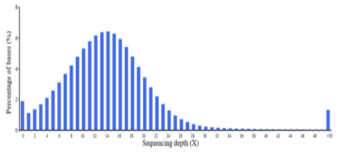	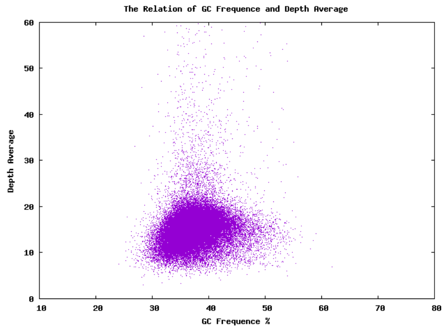

S209

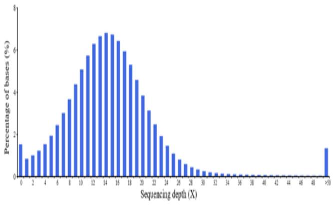

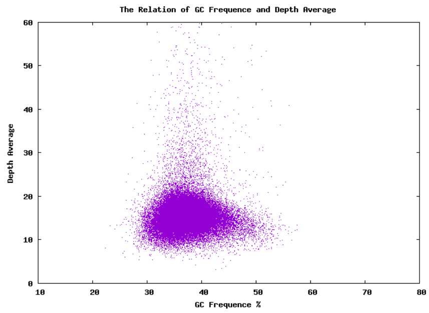

S159

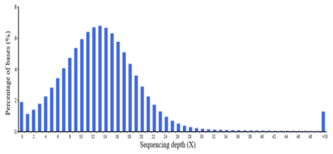

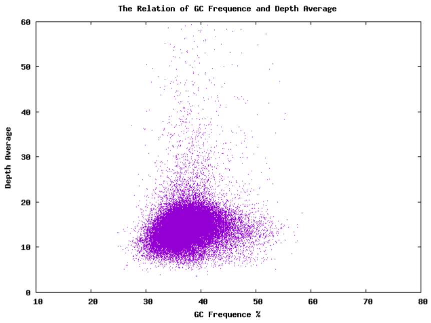

S200

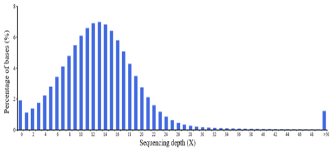

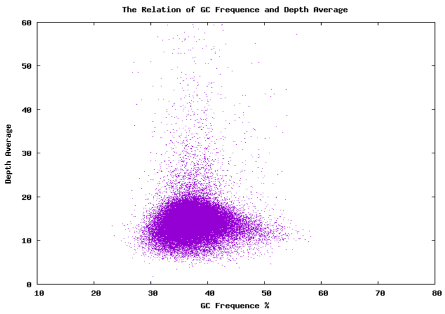

P. davidiana

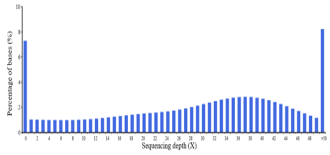

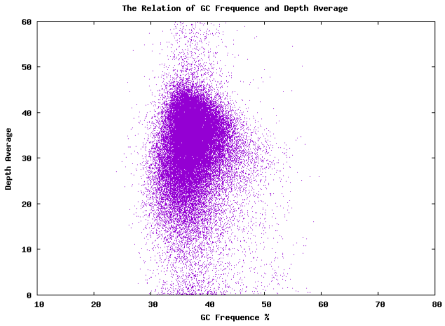

Supplementary Table 4. Summary of assembly results for individual accessions for pan-genome establishment

SampleID	Total length (bp)	Scaffold N50 (bp)	Contig N50 (bp)	GC content (%)
P. mume Ref	232,824,624	24,358,621	32,607	37.46
435	213,789,697	23,222	15,510	37.61
159	213,274,876	20,516	13,942	37.64
176	208,167,184	23,448	16,128	38.05
1	215,741,086	19,726	13,737	37.62
200	211,528,546	21,975	14,682	37.37
209	208,425,300	32,045	19,783	37.38
248	219,683,305	17,415	12,260	37.51
89	225,950,022	27,965	19,732	37.53
93	210,869,098	21,256	14,109	37.58
P. salicina	210,250,286	14,669	10,307	37.19
P. davidiana	237,165,143	21,978	15,674	37.43
P. sibirica	217,955,667	26,496	19,703	37.29
P. persica	227,252,106	26,807,724	214,242	37.51

Supplementary Table 5. Summary of gene prediction and assembly-based SNP detection for each pan-genome sample

Sample ID	No. of SNPs	Annotation		
		Gene number	Function	% of annotated
435	662,804	25,712	23,021	89.53
159	----	25,926	23,265	89.74
176	1,304,100	25,645	22,929	89.41
1	1,316,808	25,533	23,027	90.19
200	1,422,453	25,484	22,903	89.87
209	1,364,373	25,575	23,033	90.06
248	1,188,372	26,593	23,387	87.94
89	1,478,080	26,754	23,666	88.46
93	1,341,577	25,521	23,031	90.24
P. salicina	3,146,510	24,294	22,199	91.38
P. davidiana	3,325,429	26,726	23,902	89.43
P. sibirica	2,856,920	26,303	23,468	89.22
P. persica	3,383,397	27,792	25,300	91.03

Supplementary Table 6. Summary of core genomes of mei and *Prunus*

Core_Samples*		core_8_cultivated		core_9_mei		core_12 (without peach)		core_13_all	
Ratio		ratio 0.8	ratio 1	ratio 0.8	ratio 1	ratio 0.8	ratio 1	ratio 0.8	ratio 1
Raw_data	length/bp	149,920,594	110,240,014	149,095,884	113,423,575	139,646,852	81,469,453	126,789,435	78,178,463
	percent	64.62%	47.52%	64.27%	48.89%	60.19%	35.12%	54.65%	33.70%
	mean/bp	1613.11	1195.96	1575.62	1185.93	1402.67	1081.7	1380.17	1120.42
Filter_500	length/bp	140,623,220	100,667,887	139,567,657	103,525,363	129,120,493	73,794,113	117,070,788	71,277,630
	percent	60.61%	43.39%	60.16%	44.62%	55.66%	31.81%	50.46%	30.72%
	mean/bp	2913.32	2121.91	2931.85	2118.12	2955.65	1991.48	2934.69	2013.55

*Different sets of core sequence are generated from 8 cultivated and one wild (S435) *Prunus mume* individuals, and four close relatives of mei, *P. sibirica*, *P. davidiana*, *P. salicina* and *P. Persica*, as listed in Supplementary Table 4.

Supplementary Table 7. Core genes extracted from core genome and by gene clustering

	Core genes from core genome	Core genes from clusters (reference genes)	Overlap	Core genes from clustering (genes not in ref*)	Total Core genes	Percentage of reference (31,390 genes)
Mei	17,636	18,077	13,214	229	22,499	71.68%
Prunus	13,181	15,596	9,642	145	19,135	60.96%

**P. mume* reference gene set

Supplementary Table 8. Summary of PAVs identification in each genome

Sample	Genome size (bp)	Length (bp) identity <95	% of genome	Length (bp) identity <90	% of genome
435	213,789,697	13,912,559	6.51	189,811	0.09
S159	208,167,184	22,766,053	10.94	389,753	0.19
S176	215,741,086	27,163,264	12.59	232,961	0.11
S1	211,528,546	26,451,119	12.50	330,057	0.16
S200	208,425,300	25,792,527	12.37	488,227	0.23
S209	219,683,305	21,780,416	9.91	277,938	0.13
S248	225,950,022	25,212,475	11.16	389,516	0.17
S89	210,869,098	35,817,873	16.99	547,599	0.26
S93	210,250,286	22,957,470	10.92	279,462	0.13
P. salicina	213,274,876	76,217,755	35.74	25,846,255	12.12
P. dacidiana	237,165,143	117,850,420	49.69	13,921,660	5.87
P. sibirica	217,955,667	90,018,666	41.30	12,759,303	5.85
P. persica,	227,252,106	138,479,165	60.94	8,935,279	3.93

Supplementary Table 9. Specific genes identified from clustering and PAV sequence analysis

Sample	Specific_genes_from cluster analysis	Specific_gene_from PAV sequence (identity <95)	Overlap*	Specific_genes_from PAV sequence (identity <90)	Overlap
435	999	1,731	145	17	1
S159	1,417	3,638	310	36	4
S176	1,238	4,182	290	18	0
S1	1,172	4,030	274	40	6
S200	1,227	4,345	288	61	9
S209	902	3,407	207	27	2
S248	1,655	4,075	365	42	9
S89	1,298	5,257	356	51	2
S93	1,093	3,556	212	29	2
P. salicina	2,295	13,840	862	1,860	207
P. davidiana	3,322	18,302	1,404	1,003	175
P. sibirica	2,514	11,366	1,075	747	125
P. persica	4,086	22,054	1,748	415	102
TOTAL	23,218	99,783	7,536	4,346	644

*overlap between specific genes identified by clustering and by PAV analysis

Supplementary Table 10. PAVs used for investigating population-specific patterns

Sample ID	scaffold_ID	Start	End	Length (bp)
89	C1628872	1	1161	1161
248	C1673286	60	4295	4236
159	scaffold10250	63	2089	2027
1	scaffold10293	206	1411	1206
209	scaffold10515	11232	13712	2481
209	scaffold10843	3241	11314	8074
176	scaffold11270	1	2123	2123
209	scaffold11379	3759	5117	1359
200	scaffold11396	12786	14038	1253
248	scaffold11419	52	2664	2613
1	scaffold11499	1165	4643	3479
1	scaffold11499	5869	7056	1188
89	scaffold11583	6233	8065	1833
248	scaffold11587	1	1752	1752
248	scaffold11587	3192	5380	2189
1	scaffold11659	142	1169	1028
200	scaffold11780	2972	7519	4548
159	scaffold11782	1231	4268	3038
159	scaffold11825	4775	6219	1445
1	scaffold12124	2838	4624	1787

1	scaffold12566	2788	5560	2773
1	scaffold12566	469	2078	1610
159	scaffold12570	680	3630	2951
1	scaffold12624	134	2341	2208
248	scaffold12865	269	1662	1394
176	scaffold13011	1600	4400	2801
248	scaffold13061	44	2689	2646
248	scaffold13132	1040	4079	3040
159	scaffold13190	313	1348	1036
248	scaffold13243	9534	11579	2046
89	scaffold13269	6226	7510	1285
1	scaffold13383	1934	4476	2543
1	scaffold13396	758	7297	6540
1	scaffold13442	4222	5637	1416
248	scaffold13691	69	1332	1264
1	scaffold13979	7129	12998	5870
176	scaffold14058	929	3314	2386
93	scaffold14188	51	1173	1123
1	scaffold14254	2701	4006	1306
1	scaffold14315	1402	4471	3070
248	scaffold14546	853	1957	1105
1	scaffold15210	4730	7337	2608
1	scaffold15210	909	4164	3256
176	scaffold15274	1	1653	1653
1	scaffold15298	1174	3199	2026
248	scaffold15305	36	1597	1562
1	scaffold15366	3544	4884	1341
1	scaffold15491	1	2425	2425
248	scaffold15637	279	2580	2302
200	scaffold15686	1	1470	1470
159	scaffold15688	238	2378	2141
1	scaffold16140	796	1931	1136
209	scaffold1649	2851	12598	9748
1	scaffold16719	1534	2592	1059
248	scaffold18754	1157	2648	1492
248	scaffold19624	1768	2772	1005
248	scaffold2018	206	1290	1085
176	scaffold2121	2751	4033	1283
1	scaffold301	5700	7548	1849
176	scaffold3610	1477	3015	1539
200	scaffold3634	1694	2711	1018
159	scaffold3644	10764	13742	2979
89	scaffold364	21296	23076	1781
89	scaffold364	24285	27768	3484

200	scaffold3882	2237	3617	1381
209	scaffold426	33296	37194	3899
209	scaffold426	41598	43042	1445
209	scaffold426	48036	49447	1412
248	scaffold4321	2895	11414	8520
89	scaffold525	8793	9826	1034
435	scaffold546	5215	6239	1025
89	scaffold5804	41366	43002	1637
248	scaffold6391	3293	5107	1815
248	scaffold6391	6018	7346	1329
209	scaffold639	12587	14494	1908
248	scaffold6580	1275	4113	2839
159	scaffold6604	1	2147	2147
159	scaffold6604	3030	4155	1126
435	scaffold6862	14131	19328	5198
435	scaffold6862	9164	10949	1786
248	scaffold6928	569	3112	2544
248	scaffold7272	14666	18067	3402
248	scaffold7272	173	6473	6301
248	scaffold7272	9006	12798	3793
1	scaffold787	1757	3382	1626
248	scaffold8690	2557	5182	2626
93	scaffold9111	3624	5445	1822
159	scaffold9252	4571	6623	2053
159	scaffold9252	7124	8464	1341
435	scaffold9407	688	2117	1430
435	scaffold9748	829	2791	1963
159	scaffold9783	1058	6322	5265
200	scaffold9984	1189	3426	2238

Supplementary Table 11. Summary of coverage of PAVs specific to the P11 subpopulation

Sample ID	Location	scaffold15274_1_1653_S176	scaffold13011_1600_4400_S176	Average
40*	Anhui	1	1	1
176*	Wuhan	1	0.99	0.995
37*	Anhui	0.99	1	0.995
128	Wuhan	1	0.98	0.99
244	Wuhan	1	0.98	0.99
179*	Nanjing	0.99	0.99	0.99
329*	Tibet	0.99	0.99	0.99
33*	Wuhan	0.99	0.99	0.99
64*	Wuhan	0.99	0.99	0.99
254*	Nanjing	0.98	1	0.99

103*	Wuhan	0.98	0.99	0.985
360*	Wuhan	0.98	0.99	0.985
39*	Wuhan	0.98	0.99	0.985
45*	Chongqing	0.98	0.99	0.985
109*	Wuhan	0.98	0.98	0.98
156	Wuhan	0.97	0.99	0.98
140	Quanzhou	0.92	0.89	0.905
177	Kunming	0.06	0.25	0.155
392	Beijing	0.14	0.049	0.0945
390	Beijing	0.12	0.049	0.0845
195	Japan	0.075	0.091	0.083
27	Wuhan	0.076	0.088	0.082
294	Japan	0.06	0.093	0.0765
3	Japan	0.062	0.077	0.0695
30	Wuhan	0.066	0.072	0.069
154	Kunming	0.087	0.05	0.0685
196	Japan	0.06	0.072	0.066
28	Japan	0.084	0.047	0.0655
110	Wuhan	0.06	0.071	0.0655
198	Japan	0.06	0.071	0.0655
270	Taizhou	0.093	0.036	0.0645
102	Sichuan	0.12	0	0.06
106	Wuhan	0.12	0	0.06
114	Japan	0.12	0	0.06
8	Wuhan	0.12	0	0.06
262	Qingdao	0.082	0.036	0.059
352	Kunming	0.079	0.039	0.059
321	Kunming	0.076	0.04	0.058
58	Wuhan	0.06	0.049	0.0545
256	Japan	0.071	0.037	0.054
155	Wuhan	0.06	0.042	0.051
204	Beijing	0.065	0.036	0.0505
171	Chengdu	0.06	0.036	0.048
318	Dalian	0.06	0.036	0.048
63	Nanjing	0.06	0.036	0.048

* individuals in P11 subpopulation

Supplementary Table 12. Summary data for the six sequenced transcriptomes

Sample	Total Raw Reads (Mb)	Total Clean Reads (Mb)	Clean Reads Q30 (%)	Clean Reads Ratio (%)	Total Mapping Ratio %	Uniquely Mapping Ratio %
MDL1A*	82.52	59.42	94.56	72.01	70.46	67.79
MDL2A	82.5	59.75	94.62	72.42	67.52	65.08
MDL3A	80.81	59.04	94.59	73.06	69.96	67.35
WYY1A*	79.21	59.12	94.63	74.63	63.46	61.42
WYY2A	79.27	59.34	94.14	74.86	66.85	64.56
WYY3A	79.27	59.43	93.29	74.96	68.73	66.34

*MDL, 'Mi Dan Lv', *Prunus mume* landraces with white flower and green calyx; WYY, 'Wu Yu Yu', *Prunus mume* landraces with red flower and red calyx.

Supplementary Table 13. Optimal model for each trait in GWAS

Trait	Optimal model
Petal number	Q+K
Petal color	Q+K
Stigma color	Q+K
Bud color	Q
Wood color	Q
Staminal filament color	Q
Pistil character	Q
Bud aperture	Q+K
Branch phenotype	Q
Calyx color	Q
