# Supplementary material for: The genetic architecture of floral traits in the woody plant Prunus mume
Source: Nat Commun. 2018 Apr 27;9:1702. doi: 10.1038/s41467-018-04093-z (PMC5923208; doi:10.1038/s41467-018-04093-z)
Supplement: Supplementary file 3 — Description of Additional Supplementary Files [file 41467_2018_4093_MOESM3_ESM.pdf]

Legends for each Supplementary Data file were as follow:

Supplementary Data 1. General resequencing information for mei and *Prunus* accessions. It includes 15 wild accessions, 333 cutivar samples and three related *Prunus* species, including *P. sibirica*, *P. davidiana* and *P. salicina*.

Supplementary Data 2. Indels and structural variants (SV) of resequenced mei and Prunus accessions.

Supplementary Data 3. 34 phenotypes characterized in all resequencing samples.

Supplementary Data 4. Inter- and intra-specific introgression analysis using three-population F3 test statistics.

Supplementary Data 5. Summary of enriched pathways of mei-specific core genes.

Supplementary Data 6. SNPs located within DEGs and associated with petal color.

Supplementary Data 7. SNPs located within DEGs and associated with stigma color.

Supplementary Data 8. SNPs located within DEGs and associated with calyx color.

Supplementary Data 9. SNPs located within DEGs and associated with bud color.

Supplementary Data 10. Results of promoter/enhancer(s) prediction for MYB108 genes.

Supplementary Data 11. SNPs located within DEGs and associated with wood color.

Supplementary Data 12. SNPs located within DEGs and associated with staminal filament color.

Supplementary Data 13. SNPs located within DEGs and associated with petal number.

Supplementary Data 14. SNPs located within DEGs and associated with pistil character.

Supplementary Data 15. SNPs located within DEGs and associated with bud aperture.

Supplementary Data 16. SNPs located within SEGs and associated with bud aperture.

Supplementary Data 17. SNPs located within DEGs and associated with branching phenotype.

Supplementary Data 18. Functional enrichment result of genes associated with 10 traits.
